# Mixtures of genotypes increase disease resistance in a coral nursery

**DOI:** 10.1038/s41598-022-23457-6

**Published:** 2022-11-11

**Authors:** Anya L. Brown, Dagny-Elise Anastasiou, Monica Schul, Sophia MacVittie, Lindsay J. Spiers, Julie L. Meyer, Carrie Manfrino, Thomas K. Frazer

**Affiliations:** 1 School of Natural Resources and Environment, University of Florida, Gainesville, FL 32611 USA; 2 Central Caribbean Marine Institute, N Coast Road E, Box 37, Little Cayman, KY3-2501 Cayman Islands; 3 Department of Soil, Water, and Ecosystem Sciences, University of Florida, Gainesville, FL 32611 USA; 4 Department of Molecular Cell Biology, University of California, Merced, Merced, CA USA; 5 Department of Fisheries and Aquatic Sciences, University of Florida, Gainesville, FL 32611 USA; 6 Florida Fish & Wildlife Conservation Commission, Fish & Wildlife Research Institute, Marathon, FL USA; 7 College of Marine Science, University of South Florida, St. Petersburg, FL 33701 USA; 8 Present Address: Department of Evolution and Ecology & Bodega Marine Lab, University of California, Davis, Bodega Bay, CA 94923 USA

**Keywords:** Ecological epidemiology, Conservation biology, Restoration ecology, Ecology, Biodiversity, Marine biology

## Abstract

Marine infectious diseases are a leading cause of population declines globally due, in large part, to challenges in diagnosis and limited treatment options. Mitigating disease spread is particularly important for species targeted for conservation. In some systems, strategic arrangement of organisms in space can constrain disease outbreaks, however, this approach has not been used in marine restoration. Reef building corals have been particularly devastated by disease and continue to experience catastrophic population declines. We show that mixtures of genotypes (i.e., diversity) increased disease resistance in the critically endangered *Acropora cervicornis*, a species that is frequently targeted for restoration of degraded reefs in the broader Caribbean region. This finding suggests a more generalized relationship between diversity and disease and offers a viable strategy for mitigating the spread of infectious diseases in corals that likely applies to other foundation species targeted for restoration.

## Introduction

Infectious diseases in marine organisms are notoriously difficult to diagnose^1^, and options for treatments in the field are limited and labor intensive^2–5^. Thus, disease spread can quickly outpace treatment and lead to population decline or loss. There is a pressing need, therefore, to develop and implement strategies that can mitigate disease spread within affected populations. A promising approach relies on harnessing the ecological and evolutionary processes that influence disease transmission within and across populations. For example, in agricultural and small-scale experiments with *Daphnia*, intraspecific genotypic diversity can constrain disease spread when genotypes with varying resistances are grouped together^6,7^. These findings have profound, although untested, implications for restoration of marine species that are vulnerable to diseases with few treatment options, like corals.

Coral reefs have been decimated by coral diseases^8–10^, particularly in the Caribbean. The highly transmissible white band disease (WBD)^11^, for example, led to the near complete collapse of dominant reef-building *Acropora cervicornis* and *Acropora palmata* coral populations^8,12^. More recently, stony coral tissue loss disease (SCTLD) threatens the existence of numerous other reef-building corals around the Caribbean^10,13,14^. While the etiology of SCTLD is still unknown, the application of antibiotics has been effective at a colony scale^15–20^, but cannot keep pace with continued infections and reinfections on the reef scale. Marked coral population declines and localized extinction have led to increased global efforts to restore degraded tropical reefs that often involve coral nurseries^21^.

Coral nurseries support the asexual propagation of fast-growing species such as the Caribbean staghorn coral, *A. cervicornis,* and provide fragments to replenish coral depauperate areas^21–23^. Multiple genotypes are reared in nurseries to support adaptation to future environmental conditions^23^. However, ocean nursery-reared corals, like wild corals, are subject to disease outbreaks^24^. Within a species, colonies vary in their innate immunity to diseases^25^, yet the role of this variability on disease spread within and across populations is poorly understood.

To evaluate how genotypic diversity affects the spread of disease, we tracked an outbreak of white band disease in a coral nursery of the endangered coral species, *A*. *cervicornis*^12^ over 5 months. We monitored 650 coral fragments attached to support structures (frames). Some frames harbored coral fragments originally from a single donor colony (single genotype) and other frames were comprised of fragments from multiple donor colonies representing different genotypes (mixture of genotypes)^26,27^. We tracked the presence of disease on each fragment, across all frames, over five months and related disease prevalence on frames to diversity (mixed vs single genotypes on a frame). We found mixtures of genotypes on frames led to resistance of the infectious white band disease in this nursery population harboring endangered *A. cervicornis*.

## Results

**Figure 1:**
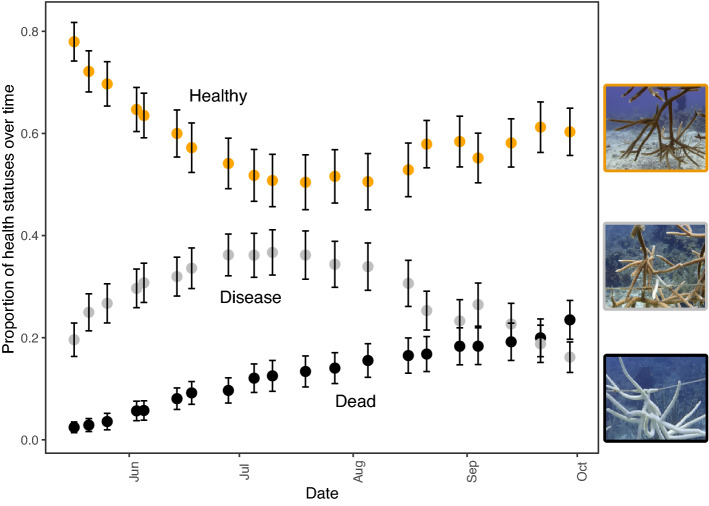
Mean ± standard error (SE) of the proportions of fragments in the nursery assigned to each health category over time. Images show the appearance of corals in each category, with healthy corals showing no apparent signs of disease (outlined in orange corresponding to the orange points); diseased corals showing sloughing of tissue and a bright white skeleton left behind the lesion (outlined in gray corresponding to the gray points); and dead corals showing bright white skeletons with no tissue present and growth of algal turf (outlined in black corresponding to the black points).

Disease prevalence peaked in mid-July (July 19), coincident with increasing water temperatures, and waned by the end of September 2019 (Figs. 1, S1).

**Figure 2:**
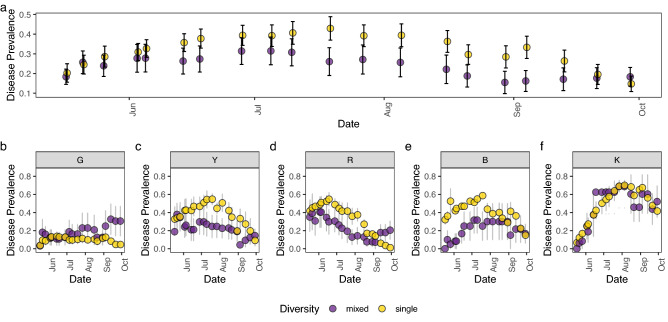
Prevalence of disease Mean ± standard error (SE) of disease prevalence across (**a**) all frames, and by genotype (**b**) “G” (**c**) “Y”, (**d**) “R”, (**e**) “B”, (**f**) “K”. Point colors represent mixed (purple) and single (yellow) genotype treatments. Genotype G (plot **b**) and Genotypes Y, R, and B (plots **c**–**e**) are the resistant and vulnerable genotypes, respectively. Genotype K (plot **f**) is highly vulnerable.

Coral colonies arranged on frames with only one genotype were significantly more likely to be diseased during the peak period July (July 19) compared to frames harboring mixed genotypes (mean ± standard error for single genotypes: 43% ± 0.06 versus mixed: 26% ± 0.07% on July 19, Fig. 2a, Model 1, Date^2^ × Diversity, p < 0.001, Table S2). Complete colony mortality was relatively low (less than 25%), although all diseased colonies showed substantial partial mortality.

Furthermore, when we compared only the genotypes found on both single and mixed frames, our results revealed intraspecific differences in disease susceptibility (Fig. 2b–f). For one genotype (G), disease prevalence was low (< 20%), regardless of whether corals were on frames with mixtures of genotypes or their own genotype, indicating that this genotype was disease resistant (Fig. 2a, Model 2, Diversity × Genotype × Date^2^ p < 0.001, Table S2). Conversely, the susceptible genotype colonies (R,Y,B) were 1.5–2× as likely to be diseased or die on single genotype frames than on mixed genotype frames (Figs. 2b–d, S3). One genotype appeared to be highly vulnerable no matter which frame type it was on (Fig. 2f, Genotype K), as it showed high disease prevalence on both single and mixed genotype frames.

## Discussion

We documented lower disease prevalence on frames with mixed genotypes compared to frames with single genotypes. We suggest intraspecific differences in susceptibility led to these emergent population and frame-level disease progression differences on mixed and single genotype frames. Because *A. cervicornis* is known to vary in susceptibility^24^ and resistance to WBD^25,28,29^, it is likely that host genotypic, immunological, or microbial variation may lead to differences in disease susceptibility and contribute to the population-level disease resistance we observed. For example, the resistant genotype (G) has been previously shown to associate with a different dominant microbial variant (*Candidatus* Aquarickettsia*)* than the susceptible genotypes (e.g., R and Y)^30^, which may play a role in differences in susceptibility, as suggested for other areas of the Caribbean^31^. Indeed, our results suggest that emergent population disease resistance is mediated by colony differences in disease resistance and susceptibility, which may, in turn, be mediated by key microbes.

Importantly, these results show that disease transmission is lowered on mixed frames, reducing disease spread to highly vulnerable individuals. Thus, some of the corals that are vulnerable to disease can be “rescued” by resistant genotypes (i.e., Fig. 2c–e), likely because resistant genotypes prevent the transmission of the disease between fragments. In fact, when the resistant genotype (G) was present on a frame, disease prevalence (both within a genotype and a frame as whole) tended to be lower than on other mixed genotype frames when the resistant genotype was absent (Figs. 2 and S2). Frames that lacked the known resistant genotype (4 mixed genotype frames) or contained the highly susceptible genotype (K) tended to have higher disease prevalence, indicating (a) disease resistance is rare, (b) there may be a threshold in which the presence of a disease resistant genotype no longer resists disease, and/or c) there are some highly susceptible genotypes that amplify disease. These hypotheses require further testing. In general, on mixed frames, corals from resistant genotypes rescue disease-vulnerable genotypes. The maintenance of the susceptible genotypes in the population allows for a broader suite of genotypes to remain in the genetic pool, which increases adaptive resilience to changing environmental conditions beyond disease^23^. Our results also highlight that because disease resistance is a cryptic and rare trait^32^, increasing genotypic diversity in nurseries increases the likelihood of diluting the disease^33^.

This finding has important implications for wild coral populations, particularly those that already exhibit low numbers of colonies and reduced genetic variability^34^. Specifically, our results suggest that the prevalence of disease will increase across the Caribbean as genotypic diversity is lost and the likelihood of the diluting effects of resistant genotypes are diminished. Residual populations although comprised of disease resistant genotypes may lack the full complement of genetic traits necessary to support selective processes that give rise to adaptation and evolution^23^.

Our work adds to the literature suggesting that diversity can reduce the spread of infectious diseases by distributing pathogens across non-viable hosts, i.e. a dilution effect^35^. Low genetic diversity has been correlated with increased disease prevalence^35–38^ for mammals^39,40^, frogs^41^, invertebrates^7,42^, and plants^6,43,44^. We suggest the linkage between diversity and disease is even more general than we have previously realized. Here, we highlight this relationship in a marine system: between an infectious disease and mixtures of genotypes in endangered corals. The ecological and evolutionary phenomena that underpin our findings for *A. cervicornis* have broader conservation consequences, and we suggest incorporating aggregations of diverse genotypes when restoring corals, and other marine species, particularly those vulnerable to infectious diseases.

## Methods

### Nursery design

We monitored 650 individual corals in an ocean-based nursery offshore of the Central Caribbean Marine Institute (CCMI) on Little Cayman Island, Cayman Islands. The nursery was at a depth of 18 m. This nursery supplies coral colonies for re-populating nearby reefs, similar to many other coral nurseries around the Caribbean and the world^21–23,45^*.* In general, nurseries rely on repeated fragmenting of corals to create numerous clones from a single donor colony. The resulting coral fragments are then suspended in the water column on structures within the nursery and allowed to grow before being placed on reefs, a process referred to as outplanting^21^. Corals in the CCMI nursery were attached to PVC frames using monofilament line that was secured with crimps. Frames were 3-m wide and 1.5-m high. The structures were anchored using ropes tied to cinderblocks and held upright by empty plastic jugs partially filled with compressed air. Frames were ~ 1–3 m away from any other frame (see Fig. S2).

Because genotypic diversity is critical for populations to adapt to changing conditions, multiple genotypes (i.e., fragments from multiple donor colonies) are grown in nurseries to enhance genetic diversity of outplanted colonies and improve resilience of local populations. The CCMI nursery was established in 2012, and it started with five fragments collected from five colonies at three locations around Little Cayman Island. After collection, the donor colonies were determined to be genetically distinct via Genotyping by Sequencing (GBS) to produce single nucleotide polymorphisms (SNPs)^26^. These colonies were color coded and denoted by abbreviations: Blue (B), Green (G), Red (R), Yellow (Y), Black (K). These colonies also show genotypic variation in growth^27^ and microbial communities (from colonies in the G, Y and R genotypes)^30^. We collected nine additional fragments from isolated colonies in nine locations in 2016. We consider these fragments different genotypes based on colony isolation and the high degree of small-scale genetic diversity reported for *A. cervicornis*^23,26,46^. Thus, the nursery was considered to contain 14 genotypes at the onset of the disease (see Table S1 for names and which frames genotypes were on).

We arranged fragments on either mixed (n = 13) or single genotype frames (n = 17), with ~ 30 cm between each fragment. Each frame contained 5–50 corals (Fig. S2, Table S1). Each of the single genotype frames was populated by one of the original 5 genotypes. Mixed frames contained 3–8 genotypes per frame. The genotypic identity of individual colonies suspended on the frames was tracked with colored beads threaded on the monofilament above the crimp used to secure the fragment on the frame (Fig. S2, Table S1). Here, we used the abbreviations of the colors for simplicity.

### Disease monitoring

Starting in spring (May) 2019, while on a routine cleaning of the nursery, divers noticed sloughing of tissue along disease fronts that progressed toward the tips of the fragments. The symptoms of the disease characterized it as an acute tissue loss syndrome that matched the descriptions of White Band Disease Type I and rapid tissue loss disease^4^, which visually are both identified as White Band Disease^47^ or WBD. We also recovered putative WBD pathogen *Vibrio harveyii* in diseased tissues^48^ (Schul et al. in review). Previously, this disease was implicated in decimating populations of the critically endangered coral, *A. cervicornis*. WBD is highly infectious, and can spread through coral-coral contact as well as through waterborne transmission^11^.

We began monitoring the disease on 17 May 2019 and ceased on 29 September 2019. Weekly, divers monitored each of the fragments in the nursery, visually assessing their health status. The health of the different fragments was recorded as: Healthy (no disease apparent), Diseased (presence of disease identified by sloughing tissue), or Dead (bright white skeleton with no tissue and presence of small filamentous turf algae, Fig. 1). When we observed recovery of diseased fragments, characterized by tissue growing onto a dead skeleton, the status of the colony was revised to “Healthy.” Across the whole monitoring period, 70% of colonies (453) in the nursery showed signs of disease during two or more sampling events. Complete coral mortality was relatively low (less than 25%), although all diseased corals showed substantial partial mortality. Nearly 500 colonies (more than 75% of the nursery) recovered or were unaffected by the disease.

## Analysis

To model the prevalence of disease on frames containing mixed vs single genotypes across all of the fragments in the nursery, we applied a mixed effects binomial model calculated in R (v 4.0.0) using glmTMBB^49^, and car packages^50^ on the counts of disease versus the counts of not diseased (healthy and dead) corals. The model treated Diversity as a fixed effect (colonies on a single or mixed genotype frame), Date (date of sampling) as a quadratic fixed effect to capture the dynamics of the disease, and a random intercept for the frame sampled (Model 1: Date^2^ × Diversity + Density + (1|Frame)) to account for the repeated sampling of fragments on frames, and effects associated with the frame. We also included an interaction term, expecting the number of diseased corals to change over time, and that this would depend on whether corals were on single versus mixed frames. We included density of corals on the frame as a fixed effect. Assumptions were tested by simulating and testing the outliers of the residuals of the binomial model (DHARMa package).

To understand the interactive effects of genotype and diversity, we also applied a model to the data for the five genotypes that were present on both single and mixed frames. This model included Diversity as a fixed effect (single vs mixed); Genotype as a fixed effect (B, R, K, Y, and G), a quadratic fixed effect for Date (Model 2: Date^2^ × Genotype × Diversity + Density + (1|Frame)), and a random intercept for the frame sampled, again accounting for the repeated sampling of the frames. We also included density as a fixed effect.

## Supplementary Information

Supplementary Information.

## Data Availability

The datasets generated during the current study are available in the Dryad repository: Brown, Anya et al. (2022), CCMI nursery coral disease 2019, Dryad, Dataset, 10.25338/B8F643. The code is available at: https://github.com/anyabrown/coral_nursery_disease_frames
