# Supplementary material for: Mixtures of genotypes increase disease resistance in a coral nursery
Source: Sci Rep. 2022 Nov 11;12:19286. doi: 10.1038/s41598-022-23457-6 (PMC9652365; doi:10.1038/s41598-022-23457-6)
Supplement: Supplementary file 1 — Supplementary Information. [file 41598_2022_23457_MOESM1_ESM.pdf]

Supplemental Information for

Mixtures of genotypes increase disease resistance in a coral nursery

Anya L. Brown[1,2*], Dagny-Elise Anastasiou[3], Monica Schul[4], Sophia MacVittie[3], Lindsay J. Spiers[5], Julie L. Meyer[6], Carrie Manfrino[3], Thomas K. Frazer[7]

[1]School of Natural Resources and Environment, University of Florida, Gainesville, FL, USA, 32611

[2]current: Department of Evolution and Ecology & Bodega Marine Lab, University of California, Bodega Bay, CA, USA, 94923

[3]Central Caribbean Marine Institute: N Coast Road E Box 37 Little Cayman KY3, 2501, Cayman Islands

[4]Department of Environmental Engineering Sciences, University of Florida, Gainesville, FL, USA, 32611

[5]Department of Fisheries and Aquatic Sciences, University of Florida: Gainesville, FL, USA, 32611

[6] Department of Soil, Water, and Ecosystem Sciences, University of Florida: Gainesville, FL, USA, 32611

[7]College of Marine Science, University of South Florida; St. Petersburg, FL, 33701

[*]Corresponding Author
anybrown@ucdavis.edu

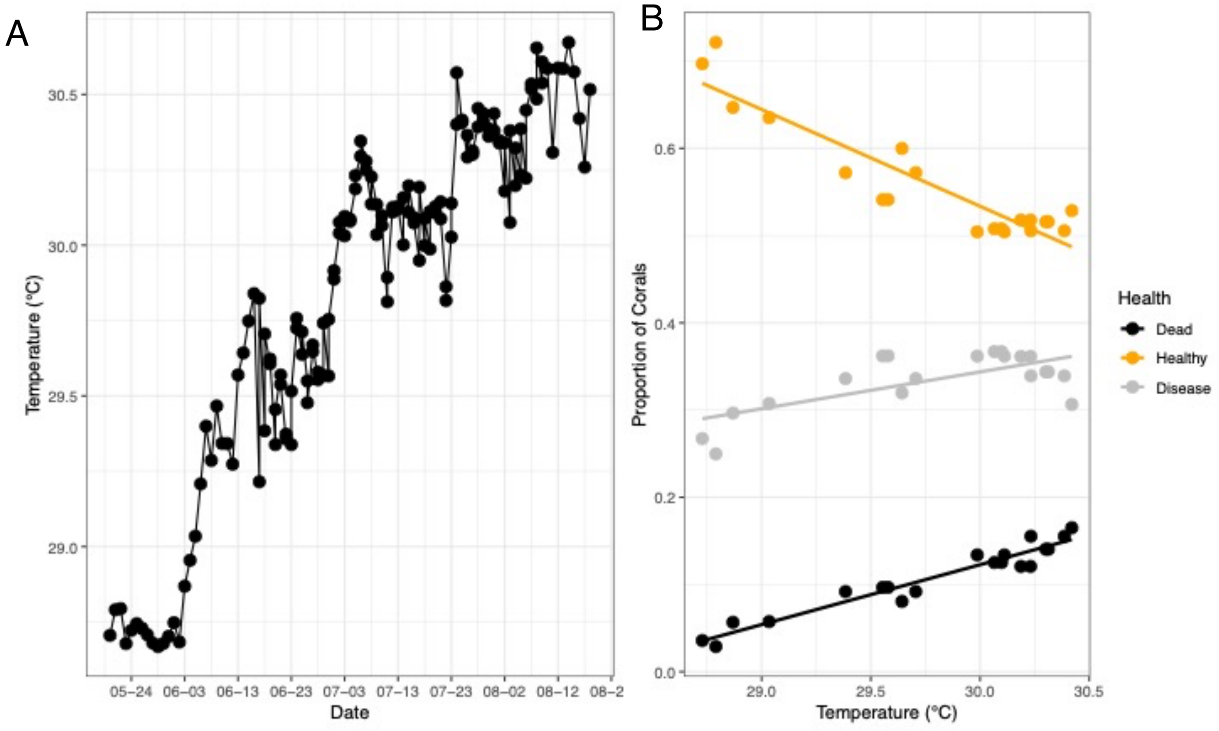

Figure S1. **Relationship between temperature and coral health category** The (A) seawater temperature (°C) over the time period that included the peak of disease in the nursery (July) in Little Cayman Island and (B) the relationships among the proportions of fragments in the three categories characterizing health and temperature in that window. Temperature data were collected with HOBO® temperature loggers on the north shore of Little Cayman Island at a depth of 18 m. The health of corals was significantly correlated with temperature based on a linear model comparing proportions of corals in each category with temperature (adjusted $R^2 = 0.99$; $F_{5, 54} = 891$; $p < 2.2*10^{-16}$). The proportions of diseased and dead corals increased with increasing temperature (Disease: y = 0.042x-0.92; Dead: y= 0.07x-1.94), and the proportion of healthy corals decreased (Healthy: y = -0.11+3.86). Note, the proportions are not independent of each other, thus neither are the slopes of the lines.

Fig. S2.

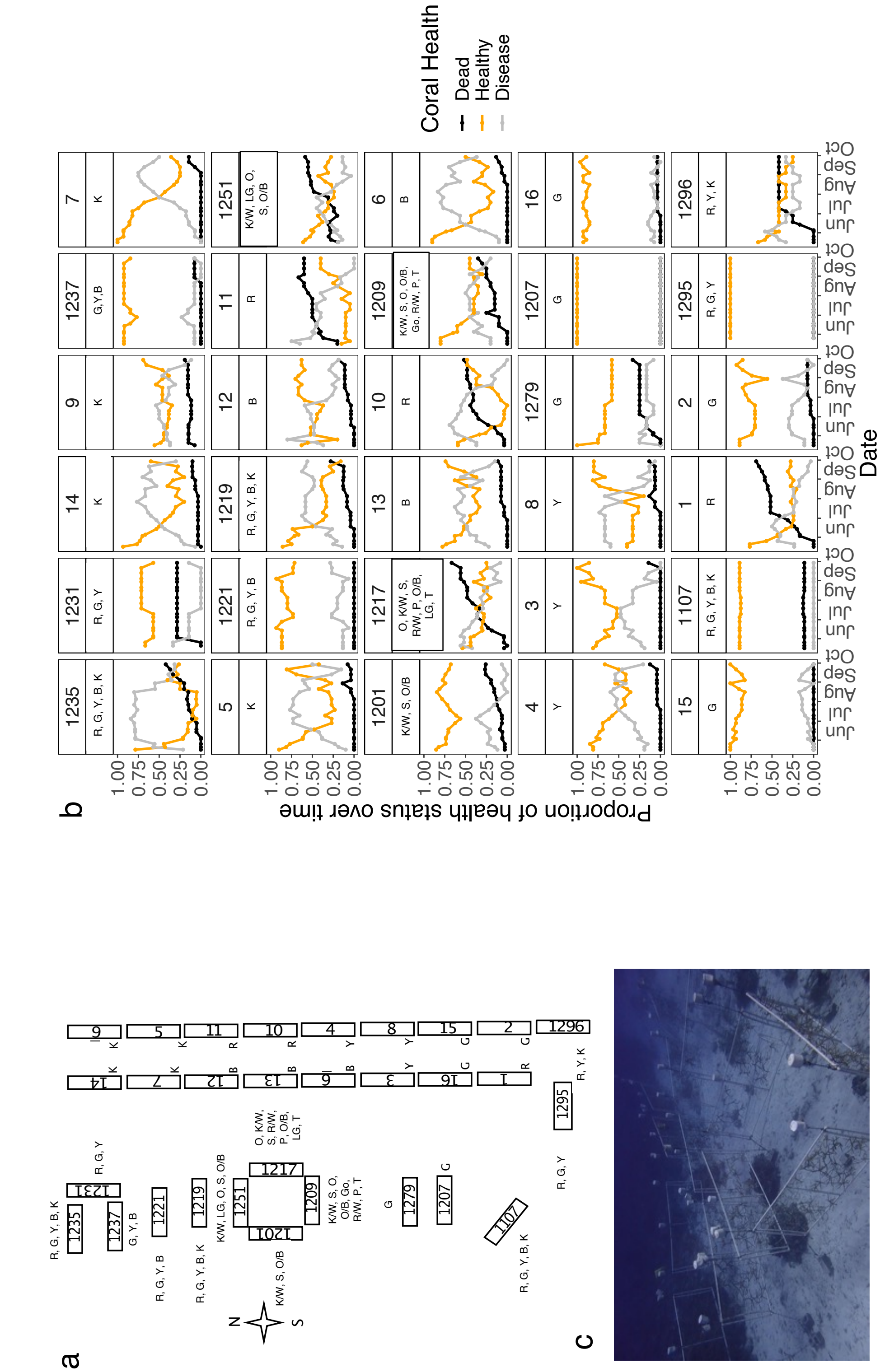

a
b
c
Coral Health
Dead
Healthy
Disease
Proportion of health status over time
Date

Figure S2. Coral nursery map and frame-level coral health Details of the nursery off Little Cayman Island, with (a) map of the nursery where each rectangle represents a frame identified by unique numbers. Letters next to the frames represent different genotypes (described in Table S1). Location and orientation of each frame represents where the frame is located in space. (b) Proportion of fragments in each category of health (orange indicates healthy, black indicates dead, and gray indicates disease) over time for each frame. Labels on the top of each plot represent the frame number and which genotypes are present. See Table S1 for number of corals and the names of the genotypes present. (c) Image of frames in the nursery (photo credit: CCMI).

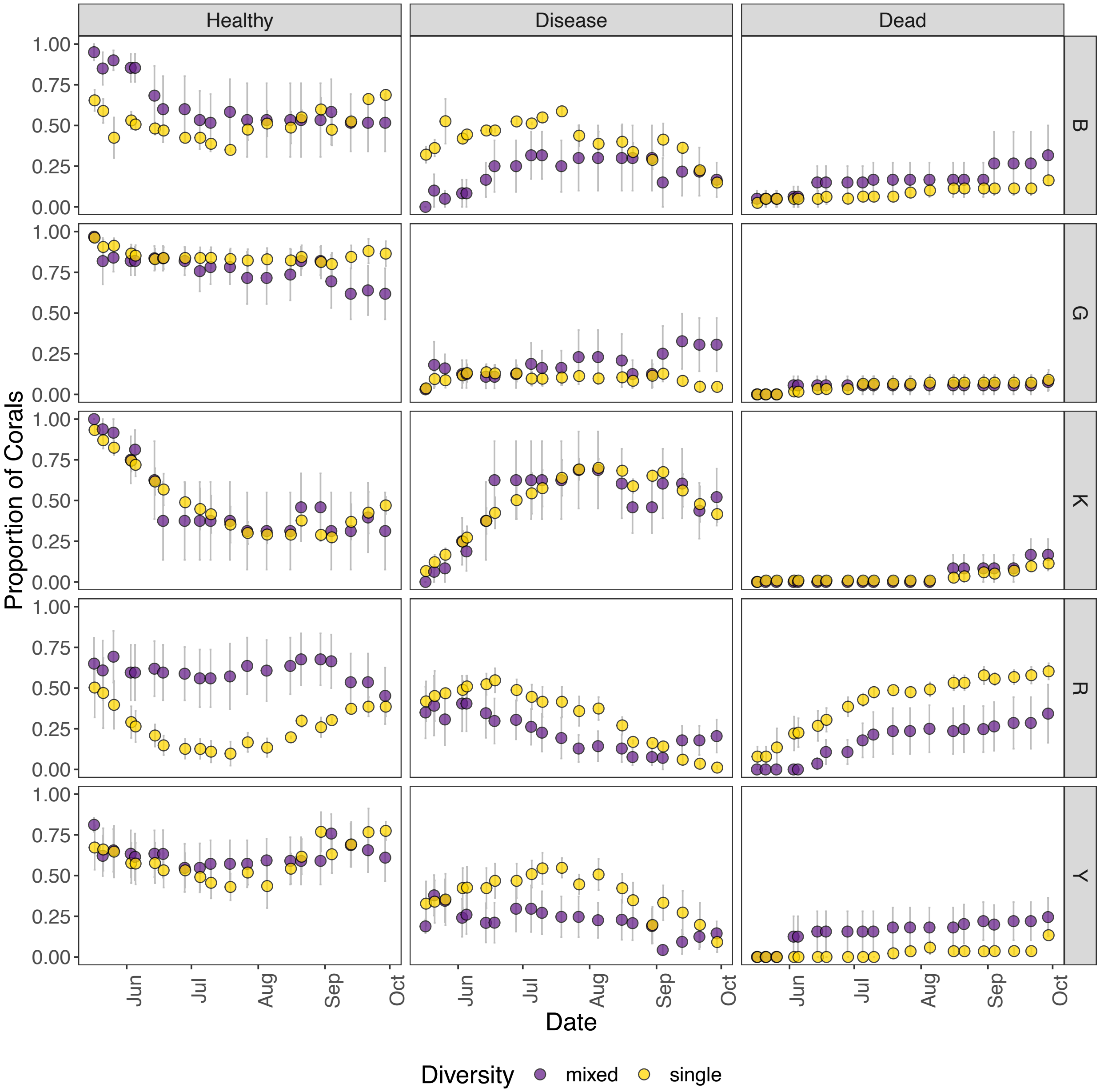

Figure S3. Proportion of corals in each health category by each genotype. Mean± standard error (SE) of proportion of corals in each of the health categories (columns: Healthy, Disease and Dead) for each of the genotypes represented on the single and mixed genotype frames (rows: B, G, K, R, Y). Colors indicate mixed (in purple) and single (yellow) genotypes on a frame.

Table S1.

Frame number	Number of corals	Genotype(s)
1	30	R
2	30	G
3	30	Y
4	30	Y
5	30	K
6	30	K
7	30	K
8	30	Y
9	30	B
10	30	R
11	30	R
12	30	B
13	30	B
14	30	K
15	30	G
16	30	G
1107	37	R, G, Y, B, K
1201	50	S, K/W, O/B
1207	5	G
1209	50	K/W, S, O, O/B, Go, R/W, P, T
1217	50	O, K/W, S, R/W, P, O/B, LG, T
1219	35	R, G, Y, B, K
1221	12	R, G, Y, B
1231	9	R, G, Y
1235	36	R, G, Y, B, K
1237	12	G, Y, B
1251	50	K/W, LG, O, S, O/B
1279	12	G
1295	12	R, G, Y
1296	30	R, Y, K

Table S1. Frame number and the number of corals on the frame, as well as the genotypes present. Letters correspond to color codes, which represent the different genotypes. R = red, G = green, Y = yellow, K = black, B = blue, K/W = black and white, S = silver, O= orange, O/B = orange and blue, Go = gold, T = turquoise, P = pearl, LG = lime green, R/W = red and white.

Table S2
Model 1:

	ChiSq	df	p-value
Diversity	2.66	1	0.103
**Date^2**	**358.71**	**2**	**< 2.2e-16**
Density	1.56	1	0.211
**Diversity* Date^2**	**23.47**	**2**	**8.00E-06**

Model 2:

	ChiSq	df	p-value
**Diversity**	**5.88**	**1**	**0.015**
**Date^2**	**15.02**	**2**	**0.0005**
**Genotype**	**16.24**	**4**	**0.002**
Density	0.32	1	0.57
**Diversity* Date^2**	**15.12**	**2**	**0.0005**
Diversity*Genotype	5.87	4	0.21
**Date^2*Genotype**	**109.97**	**8**	**< 2.2e-16**
**Diversity* Date^2*Genotype**	**39.13**	**8**	**4.66E-06**

Table S2. Analysis of Deviance table showing Type III Wald tests for the two binomial models. Model 1 is: Date^2 x Diversity + Density+ (1|Frame), and Model 2 is: Date^2 x Genotype x Diversity + Density + (1|Frame). Terms in bold are significant.
